# Effects of Caffeinated and Decaffeinated Coffee Consumption on Metabolic Syndrome Parameters: A Systematic Review and Meta-Analysis of Data from Randomised Controlled Trials

**DOI:** 10.3390/medicina57090957

**Published:** 2021-09-11

**Authors:** Nur Nadiah Syuhada Ramli, Areej A. Alkhaldy, Abbe Maleyki Mhd Jalil

**Affiliations:** 1 School of Nutrition & Dietetics, Faculty of Health Sciences, Universiti Sultan Zainal Abidin, Kuala Nerus 21300, Terengganu, Malaysia; nadiahsyuhada1996@gmail.com; 2 Clinical Nutrition Department, Faculty of Applied Medical Sciences, King Abdulaziz University, P.O. Box 80215, Jeddah 21589, Saudi Arabia; aalkhaldy@kau.edu.sa

**Keywords:** metabolic syndrome, caffeinated coffee, decaffeinated coffee, green coffee extract, chlorogenic acid

## Abstract

Coffee is rich in phenolic acids, such as caffeic acid and chlorogenic acid (CGA). Polyphenol-rich diets were shown to reduce the risk of metabolic syndrome (MeTS). *Background and Objectives*: This systematic review and meta-analysis discusses the effects of coffee consumption and its dose-response on MeTS parameters. *Materials and Methods*: PubMed and Scopus^®^ were searched for relevant articles published between 2015 and 2020. This review focused on randomised controlled trials (RCTs) investigating the effect of coffee consumption on anthropometric measurements, glycaemic indices, lipid profiles, and blood pressure. Data from relevant studies were extracted and analysed using random, fixed, or pooled effects models with 95% confidence intervals (CIs). *Results*: Green coffee extract (GCE) supplementation (180 to 376 mg) was found to reduce waist circumference (weighted mean difference (WMD) = −0.39; 95% CI: −0.68, −0.10), triglyceride levels (WMD = −0.27; 95% CI: −0.43, −0.10), high−density lipoprotein−cholesterol levels (WMD = 0.62; 95% CI: 0.34, 0.90), systolic blood pressure (WMD = −0.44; 95% CI: −0.57, −0.32), and diastolic blood pressure (WMD = −0.83; 95% CI: −1.40, −0.26). Decaffeinated coffee (510.6 mg) reduced fasting blood glucose levels (WMD = −0.81; 95% CI: −1.65, 0.03). The meta-analysis showed that the intake of GCE containing 180 to 376 mg of CGA (administered in a capsule) and liquid decaffeinated coffee containing 510.6 mg of CGA improved the MeTS outcomes in study participants. *Conclusions*: The findings of the review suggested that the effect of coffee on MeTS parameters varies depending on the types and doses of coffee administered. A more detailed RCT on specific coffee doses (with adjustment for energy and polyphenol intake) and physical activity is needed to further confirm the observed outcomes.

## 1. Introduction

Metabolic syndrome (MeTS) is a cluster of complex metabolic disorders [1] characterised by the presence of any three of the following five medical conditions: abdominal obesity, high serum triglyceride (TG) levels, low high-density lipoprotein cholesterol (HDL-c) levels, elevated blood pressure, and elevated fasting blood glucose (FBG) levels [2]. The global prevalence of MeTS is approximately 3.3% (range, 0%–19.2%), with a prevalence of 11.9% (range, 2.8%–29.3%) in children with obesity and 29.2% (range, 10%–66%) in adults with obesity [3]. According to estimates, 12%–37% and 12%–26% of the population in Asia and Europe, respectively, are affected by MeTS [4]. Genetic and lifestyle-related factors, such as alcohol intake, smoking, sedentary habits, and poor dietary habits, such as intake of sugar-sweetened beverages, were identified as risk factors in MeTS development. Dietary interventions have helped control and improve MeTS parameters and, hence, are considered to be the most effective preventive strategy for MeTS [5].

Coffee (*Coffea* spp., *Coffea arabica*, *Coffea robusta*, and *Coffea liberica*) is one of the most popular beverages worldwide, with an estimated consumption of 500 billion cups per year [5]. Bioactive compounds in coffee, such as chlorogenic acid (CGA), caffeine, niacin, and magnesium, may play a role in reducing the risk of type 2 diabetes mellitus (T2DM) and liver disease [6]. A previous study suggested that CGA may improve the antioxidant status and reduce low-density lipoprotein cholesterol oxidation, whereas caffeine may slow the inflammation process, thereby providing protection against free radical formation and preventing endothelial damage [7]. Meanwhile, a study showed inconsistent results on the association between coffee consumption and the risk of MeTS. The study suggested that CGA may increase the total plasma homocysteine content, whereas caffeine may increase blood pressure by stimulating the sympathetic nervous system [8].

However, even though coffee consumption and chronic diseases (e.g., T2DM and cardiovascular diseases) were investigated in several studies, the association between the intake of either caffeinated or decaffeinated coffee and MeTS remains inconclusive. Previous studies showed that ground and instant caffeinated coffees significantly increased energy expenditure (3 to 12 h post ingestion) compared with decaffeinated coffee or placebo [9,10]. Early studies showed that caffeine, ground caffeinated coffee, and instant caffeinated coffee increased lipolysis compared with decaffeinated coffee [11,12,13,14]. Another study showed that acute caffeine ingestion increased glucose tolerance, while regular decaffeinated coffee decreased glucose tolerance compared with placebo (dextrose) [15]. However, there is limited evidence to suggest a link between caffeinated and decaffeinated coffee intake and disease outcomes in patients with MeTS in an experimental study design. This systematic review investigated the dose-dependent effects of caffeinated and decaffeinated coffee consumption on MeTS outcomes. The review will provide new empirical evidence on the effect of regular caffeinated and decaffeinated coffee consumption on metabolic syndrome parameters.

## 2. Materials and Methods

### 2.1. Eligibility Criteria

Free-living men and women (aged from 18 to 70 years) with MeTS, who did not take any medications, vitamins, and/or supplements during the study period, were selected. Participants with dietary restrictions or conditions other than MeTS and women who were pregnant or lactating were excluded.

The data from randomised controlled trials (RCTs) that investigated the effects of coffee consumption were reviewed. RCTs were chosen as they are considered to form the foundation of clinical research on interventions. The outcomes measured were waist circumference, FBG levels, TG levels, HDL-c levels, systolic blood pressure (SBP), and diastolic blood pressure (DBP). Only studies published between 2015 and 2020 and full-text articles published in English were included in this review. Studies that were published in languages other than English were excluded to avoid potential bias resulting from the poor translation of information. Animal and in vitro studies were also excluded. 

### 2.2. Search Strategy

This review was performed in accordance to the PRISMA (Preferred Reporting Items for Systematic Reviews and Meta-Analyses) guidelines. PubMed (U.S. National Library of Medicine and National Institutes of Health) and Scopus^®^ (Elsevier B.V., Amsterdam, The Netherlands) were used for this systematic review. Boolean operators were included in the keyword searches of the two electronic databases. The main keywords used were “MeTS terminology” (keyword 1), “MeTS outcome” (keyword 2), and “type of coffees” (keyword 3). The search strategy was based on two clusters: cluster 1—keyword 1 AND keyword 3; and cluster 2—keyword 2 AND keyword 3. The key search terms for MeTS terminology (keyword 1) were “metabolic syndrome”, “metabolic syndrome X”, “insulin resistance syndrome X”, “metabolic X syndrome”, “dysmetabolic syndrome X”, and “metabolic cardiovascular syndrome”. The key search terms for MeTS outcomes (keyword 2) were “abdominal obesity OR visceral obesity OR central obesity OR abdominal fat”, “blood lipid profiles OR triglycerides OR triacylglycerols OR HDL-c”, “high cholesterol OR hypercholesterolemia OR hypercholesterolaemia OR elevated cholesterol OR dyslipidemia OR dyslipidemia OR dyslipoproteinemia OR dyslipoproteinaemia OR hyperlipidemia OR hyperlipidaemia”, “hypertension OR high blood pressure OR systolic and diastolic pressure OR hypertensive”, and “hyperglycemia OR hyperglycaemia OR glucose intolerance OR impaired glucose intolerance OR fasting blood glucose”. The key search terms for the types of coffees (keyword 3) were “Arabica coffee OR *Coffea arabica*”, “Robusta coffee OR *Coffea robusta*”, “caffeinated coffee”, “decaffeinated coffee”, “filtered coffee”, “unfiltered coffee”, “espresso”, “americano”, “cappuccino”, “latte”, “macchiato”, and “mocha”.

### 2.3. Data Management and Analysis

All articles were uploaded in the Mendeley referencing software, and duplicate articles were removed using the “remove duplicate” function. Two reviewers independently screened the titles and abstracts based on the abovementioned predefined criteria. Full-text articles were reviewed for eligibility, irrelevant publications were excluded, and only the studies that met the inclusion criteria were included in the qualitative and quantitative analyses.

### 2.4. Evaluation of Studies and Data Synthesis

The mean intergroup differences and percentage reduction, which compared the values in the intervention group to baseline, were calculated for waist circumference, FBG levels, TG levels, HDL-c levels, SBP, and DBP. To calculate percentage reduction and increment, the following formula was applied: percentage reduction or increment = [final reading − baseline/baseline] × 100.

For the meta-analysis, an online calculator was used to calculate the effect size (Cohen’s d) based on the mean differences and standard deviation (SD) for each MeTS outcome between the intervention and control groups [16]. The effect size between groups was considered small (0.2), medium (0.5), or large (0.8). The standard error of the mean (SE) for each outcome measure was calculated using the following formula: SE = es/√ (es×*n*), where “es” represents the effect size. Studies for which the effect size or SD was not stated or could not be calculated were excluded from the meta-analysis. Cochran’s Q and *I*^2^ were calculated automatically using Excel worksheets [17] after inserting the effect size and SE of the mean. Cochran’s Q was used to confirm heterogeneity among data, whereas the *I*^2^ statistic was used to measure the heterogeneity level. A negative *I*^2^ value was considered equivalent to zero (indicating that the data were homogenous), whereas *I*^2^ values of 25%, 50%, and 75% were considered to correspond to low, medium, and high heterogeneity levels, respectively [18]. The fixed effects model was selected for low *I*^2^ values (<50%), whereas the random effects model was selected for high *I*^2^ values (>50%). The mean effect size data were statistically pooled in the meta-analysis and presented in a forest plot.

The risk of bias in each study was assessed using the Jadad scale. The scale was used to assess the studies on the basis of randomisation, double blinding, drop-out, and withdrawals [19]. The highest possible score obtained with this scale is five, which indicates a low potential for reporting bias.

## 3. Results

### 3.1. Study Selection

**Figure 1:**
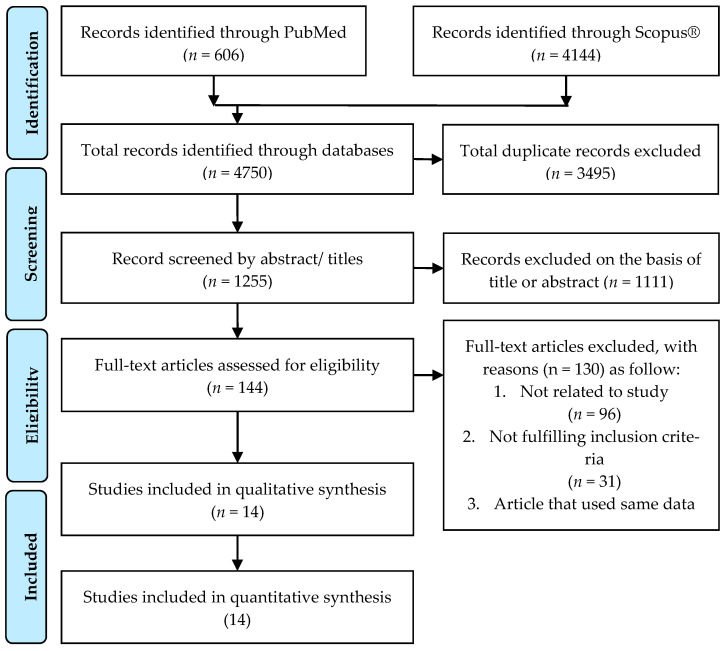
PRISMA flowchart and search strategy.

Figure 1 shows the study selection process based on the PRISMA search strategy. A total of 4750 studies were identified through PubMed (*n* = 606) and Scopus^®^ (*n* = 4144).

### 3.2. Study Characteristics

Table 1 shows the procedure of the selection of 19 RCTs (14 articles) published between 2015 and 2020 [20,21,22,23,24,25,26,27,28,29,30,31,32,33]. The number of participants in each trial (sample size, n) ranged from 10 to 142, with a total study population of 821. Five studies were conducted on apparently healthy and/or overweight individuals with obesity; other studies were conducted on individuals who were overweight or had obesity, dyslipidaemia, hypertension, or insulin resistance. Three types of coffees were used in the RCTs: caffeinated, decaffeinated, and green coffee extract (GCE) (considered as a type of decaffeinated coffee) (Table 2). Caffeinated coffee contains 5 mg of caffeine per kg body weight to 69.12 mg of caffeine per person per day (CGA content of 45.4 mg) in powdered form and 80 mg of caffeine in a volume of 250 mL. Decaffeinated coffee, with a volume of 180–400 mL, contains 369 to 780 mg of CGA per day. Green coffee extract (GCE), in a range from 10 to 1000 mg, contains 180 to 500 mg of CGA (either in capsule or tablet form).

### 3.3. Risk of Biased Based on Jadad Scale

Table 3 shows the risks of bias based on randomisation, double blinding, and drop-outs in the RCTs [19]. Most studies showed a low risk of bias, with a score of 3 or greater. Two studies scored less than 2.5, indicating a high risk of bias.

### 3.4. Summary of Systematic Review and Meta-Analysis

The outcomes evaluated in this review were waist circumference, FBG levels, TG levels, HDL-c levels, SBP, and DBP. Fourteen RCTs with 821 participants were included in the meta-analysis. Three studies investigated two interventions each (with different doses of coffee) and were considered separately in the analyses [23,29,33]. Sarria et al. investigated two groups (normocholesterolaemia and hypercholesterolaemia), and the data from the two groups were treated as findings from two different studies [22].

#### 3.4.1. Effect of Coffee on Waist Circumference

Eight studies investigated the effect of caffeinated coffee (*n* = 1), decaffeinated coffee (*n* = 3), and GCE (*n* = 4) on waist circumference (Table 1). GCE intake significantly reduced waist circumference by 1.3% to 3.0%, whereas caffeinated and decaffeinated coffee reduced the waist circumference by 0.3% and 0.4% to 1.6%, respectively. However, as shown by Sarria et al., decaffeinated coffee increased the mean waist circumference by 0.7% in normocholesterolaemic participants [22].

**Figure 2:**
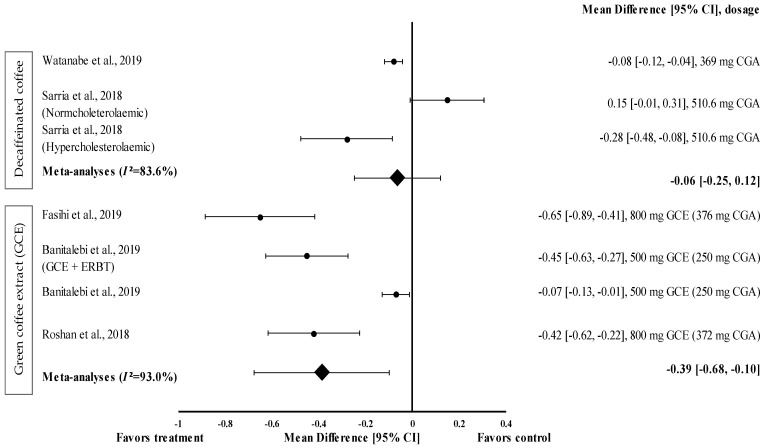
Forest plot showing the effect of decaffeinated coffee and GCE on waist circumference, expressed as mean differences between the values obtained from the intervention and control groups. A negative effect size indicates that decaffeinated coffee and GCE supplements reduce waist circumference. Meanwhile, a positive effect size indicates that decaffeinated coffee increases waist circumference. Horizontal lines represent 95% CIs. Diamonds represent the pooled effect size from the random effect analysis. CGA: chlorogenic acid, CI: confidence interval, ERBT: elastic resistance band training, GCE: green coffee extract. The values ± 0.2, ± 0.5 and ± 0.8 represent small, medium, and large effect sizes [22,26,29,30,31].

Two studies with three trials were included in the meta-analysis investigating the effect of decaffeinated coffee intake on waist circumference. The data from these studies showed a high level of heterogeneity (*I*^2^ = 83.6%) and, hence, were analysed using random effects analysis. Decaffeinated coffee showed a small effect size on mean waist circumference reduction, with d ranging from −0.08 to −0.28 (Figure 2). Two out of three decaffeinated coffee interventions led to body weight reduction (treatment group favoured). Decaffeinated coffee containing 510.6 mg of CGA showed the greatest effect size in hypercholesterolaemic participants (d = −0.28, 95% CI: −0.48, −0.08), followed by that containing 369 mg of CGA (d = −0.08, 95% CI: −0.12, −0.04). However, Sarria et al. showed that the mean waist circumference increased (d = 0.15, 95% CI: −0.01, 0.31) after decaffeinated coffee intake (favoured control group) [22]. The pooled effect size from the meta-analysis was d = −0.06 (95% CI: −0.25, 0.12) (Figure 2).

GCE supplementation tended to reduce waist circumference with a small (d = −0.07) to moderate effect size (d = −0.65) (Figure 2) and reduced the waist circumference of participants in all interventions (treatment group favoured). GCE containing 376 mg of CGA (d = −0.65, 95% CI −0.89, −0.41) showed the greatest effect size, followed by that containing 250 mg of CGA (administered along with elastic resistance band training (ERBT)) (d = −0.45, 95% CI: −0.63, −0.27), 372 mg of CGA (d = −0.42, 95% CI: −0.62, −0.22), and 250 mg of CGA (d = −0.07, 95% CI: −0.13, −0.01). The data from the studies showed a high level of heterogeneity (*I*^2^ = 93%) and, hence, were analysed using random effects analysis. The meta−analysed pooled effect size of GCE supplementation was d = −0.39 (95% CI: −0.68, −0.10) (Figure 2).

#### 3.4.2. Effect of Coffee on FBG Levels

Twelve interventions investigated the effect of caffeinated coffee (*n* = 2), decaffeinated coffee (*n* = 3), and GCE (*n* = 7) on FBG levels. GCE induced the highest percentage FBG reduction (1.1% to 14.8%), followed by decaffeinated coffee (0.8% to 4.9%). In contrast, Beam et al. showed that GCE supplementation increased the FBG levels by 8.3% compared with baseline, although not significantly [28] (Table 1).

**Figure 3:**
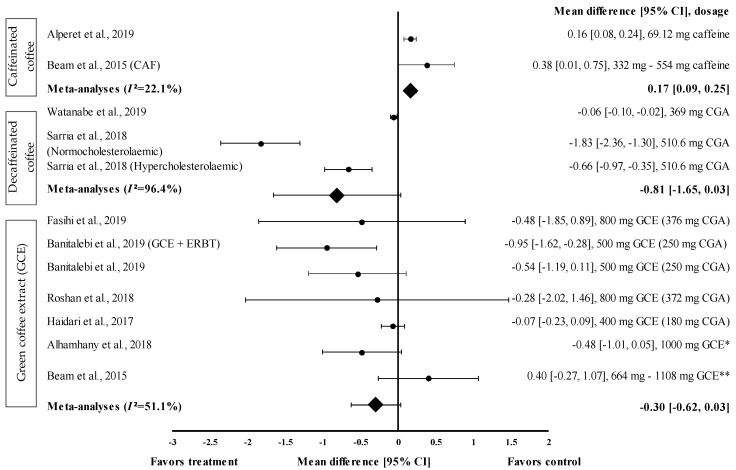
Forest plot showing the effect of caffeinated coffee, decaffeinated coffee, and GCE on FBG levels, expressed as mean differences between the values obtained from the intervention and control groups. A negative effect size indicates that decaffeinated coffee and GCE supplements reduce FBG levels. Meanwhile, a positive effect size indicates that caffeinated coffee and GCE supplements increase FBG levels. Horizontal lines represent 95% CIs. Diamonds represent the pooled effect size from random effects analysis. CAF: caffeine, CGA: chlorogenic acid, CI: confidence interval, ERBT: elastic resistance band training, FBG: fasting blood glucose, GCE: green coffee extract. The values ± 0.2, ± 0.5 and ± 0.8 represent small, medium, and large effect sizes. * CGA dose not specified, ** CGA content: 332–554 mg [20,21,22,25,26,28,29,30,31].

Nine studies with 12 trials investigating the effects of caffeinated and decaffeinated coffee on FBG levels were included in the meta-analysis. Caffeinated and decaffeinated coffee showed a small effect size by increasing the mean FBG levels, with d = 0.16 and 0.38, respectively (Figure 3). Caffeinated coffee intake (*n* = 2) increased the FBG levels (control group favoured). The greatest effect size was observed at a caffeine intake of 5 mg/kg body weight (approximately 332 to 554 mg) (d = 0.38, 95% CI: 0.01, 0.75), followed by 69.12 mg of caffeine intake (d = 0.16, 95% CI: 0.08, 0.24). The data showed low levels of heterogeneity (*I*^2^ = 22.1%) and, hence, were subjected to fixed effects analysis. The meta-analysed pooled effect size of caffeinated coffee was d = 0.17 (95% CI: 0.09, 0.25) (Figure 3).

Figure 3 shows the effect size of decaffeinated coffee intake on the reduction of FBG levels; small to very large effect sizes were observed (d = −0.06 and d = −1.83). All studies showed that the treatment group was favoured (reduced FBG levels). After the intake of decaffeinated coffee (containing 510.6 mg of CGA), normocholesterolaemic participants showed a considerably larger effect size than hypercholesterolaemic participants, with d equal to −1.83 (95% CI: −2.36, −1.30) and −0.66 (95% CI: −0.97, −0.35), respectively. A lower dose of decaffeinated coffee containing 369 mg of CGA in overweight participants led to a smaller effect size, with d = −0.06 (95% CI: −0.10, −0.02). The data showed a high level of heterogeneity, with *I*^2^ = 96.4%, and, hence, were subjected to random effects analysis. The meta-analysed pooled effect size of decaffeinated coffee was d = −0.81 (95% CI: −1.65, 0.03) (Figure 3).

Six studies with seven trials were included in the meta-analysis to investigate the effect of GCE supplementation on FBG levels. Figure 3 shows the effect size on mean FBG level reduction with small to large effect sizes (d = −0.07 and −0.95). Six out of seven interventions with GCE reduced the FBG levels compared with the baseline. GCE containing 250 mg of CGA combined with ERBT showed the greatest effect size (d = −0.95, 95% CI: −1.62, −0.28), followed by GCE containing 250 mg of CGA (GCE intake only), GCE containing 376 mg of CGA, 1000 mg of GCE (CGA dose unspecified), GCE containing 372 mg of CGA, and GCE containing 180 mg of CGA (d = −0.54, 95% CI: −1.19, 0.11; d = −0.48, 95% CI: −1.85, 0.89; d = −0.48, 95% CI: −1.01, 0.05; d = −0.28, 95% CI: −2.02, 1.46; and d = −0.07, 95% CI: −0.23, 0.09, respectively). One trial reported a null effect on FBG level reduction (favoured control group). Beam et al. showed that CGA supplementation (at 332 to 554 mg/person/day) increased the mean FBG levels, with d = 0.40 (95% CI: −0.27, 1.07) [28]. The meta-analysed pooled effect size of GCE supplementation was d = −0.30 (95% CI: −0.62, 0.03), with moderate heterogeneity of *I*^2^ = 51.1% (random effects analysis) (Figure 3).

#### 3.4.3. Effect of Coffee on TG Levels

The effect of coffee intake on TG levels was evaluated on short-term (60–120 min) and long-term (8–24 weeks) bases. Data from three short-term interventions on the effects of decaffeinated coffee on TG levels were analysed. Additionally, data from fourteen long-term interventions on the effect of coffee on TG levels (caffeinated coffee (*n* = 1), decaffeinated coffee (*n =* 7), and GCE (*n* = 6)) were analysed. Decaffeinated coffee intake increased the mean TG levels, with the increase ranging from 43.8% to 60.6%. GCE was the most effective in reducing TG levels compared to baseline, with a percentage reduction ranging from 2.2% to 11.3% (Table 1).

**Figure 4:**
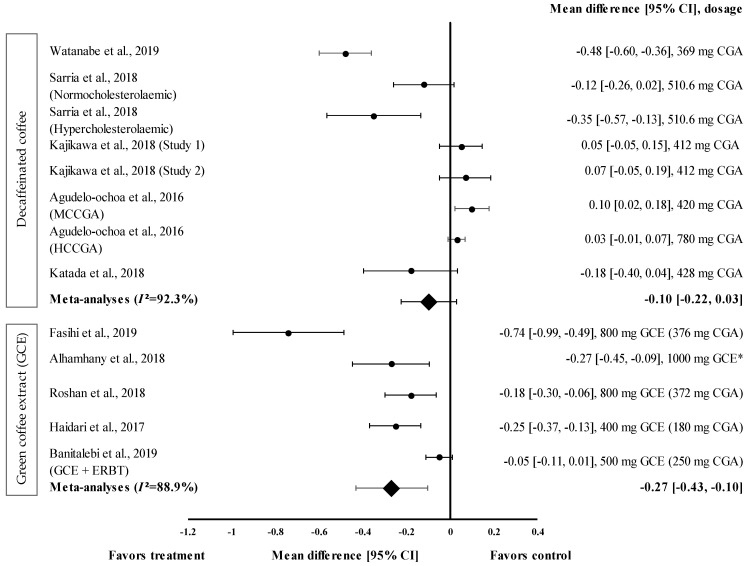
Forest plot showing the effect of decaffeinated coffee and GCE on TG levels, expressed as mean differences between the values obtained in the intervention and control groups. A negative effect size indicates that decaffeinated coffee and GCE supplements reduce TG levels. Meanwhile, a positive effect size indicates that decaffeinated coffee increases TG levels. Horizontal lines represent the 95% CIs. Diamonds represent the pooled effect size from the random effect analysis. CGA: chlorogenic acid, CI: confidence interval, ERBT: elastic resistance band training, GCE: green coffee extract, HCCGA: high CGA content, MCCGA: medium CGA content, TG: triglyceride. The values ± 0.2, ± 0.5, and ± 0.8, represent small, medium, and large effect sizes, respectively. * CGA dose not specified [21,22,23,26,29,30,31,32,33,34].

Overall, five studies with eight trials were included in the meta-analysis (Figure 4). Mean TG level reduction showed a small effect size with the d value ranging from 0.03 to −0.48 (Figure 4). Four out of eight decaffeinated coffee interventions showed the reduction of TG levels (treatment group favoured). Decaffeinated coffee containing 369 mg of CGA showed the greatest effect size (d = −0.48, 95% CI: −0.60, −0.36), followed by that containing 510.6 mg of CGA (in hypercholesterolaemic participants), 428 mg of CGA, and 510.6 mg of CGA (normocholesterolaemic participants) (d = −0.35, 95% CI: −0.57; −0.13, d = −0.18, 95% CI: −0.40, 0.04; and d = −0.12, 95% CI: −0.26, 0.02, respectively). Four trials showed a null effect on the mean TG level reduction (favoured control group). Decaffeinated coffee with CGA content ranging from 412 to 780 mg showed an effect size ranging from d = 0.03 (95% CI: −0.01, 0.07) to d = 0.10 (95% CI: 0.02, 0.18). The data showed a high level of heterogeneity with *I*^2^ = 92.3% and, hence, were subjected to random effects analysis. Decaffeinated coffee showed small pooled effect size on TG levels, with d = −0.10 (95% CI: −0.22, 0.03) (Figure 4).

GCE intake (in five trials) showed small to large effect sizes on TG levels, with d = −0.05 and d = −0.74, respectively (Figure 4). All GCE interventions reduced the TG levels (treatment group favoured). GCE containing 376 mg of CGA showed the greatest effect size (d = −0.74, 95% CI: −0.99, −0.49), followed by 1000 mg of GCE (CGA dose not specified) (d = −0.27, 95% CI: −0.45, −0.09), and GCE containing 180, 372, and 250 mg of CGA (CGA + ERBT) (d = −0.25, 95% CI: −0.37, −0.13; d = −0.18, 95% CI: −0.30, −0.06; d = −0.05, 95% CI: −0.11, 0.01; respectively). Overall, the meta−analysis showed that GCE supplementation had a small pooled effect size with a high level of heterogeneity on the mean reduction in TG levels (pooled effect size of −0.27, 95% CI: −0.43, −0.10; *I*^2^ = 88.9%) (Figure 4).

#### 3.4.4. Effect of Coffee on HDL-c Levels

Fourteen interventions reported the effect of coffee (caffeinated coffee (*n* = 1), decaffeinated coffee (*n* = 7), and GCE (*n* = 6)) on HDL-c levels. GCE caused the greatest increase in HDL-c levels, with the percentage of increase ranging from 2.4% to 15.6% (Table 1).

**Figure 5:**
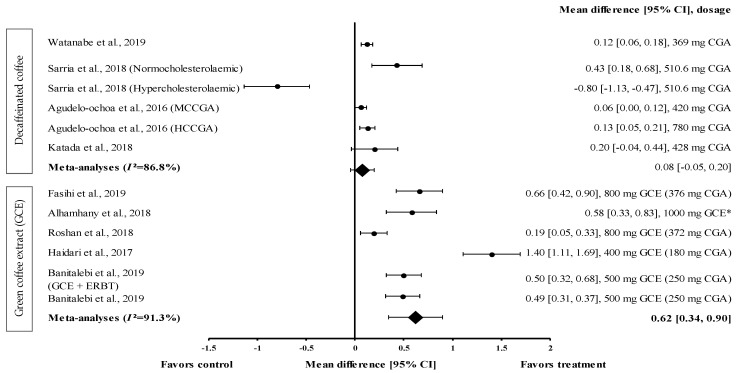
Forest plot showing the effect of decaffeinated coffee and GCE supplements on HDL-c levels, expressed as mean differences between the values obtained in the intervention and control groups. A positive effect size indicates that decaffeinated coffee and GCE supplements increased the HDL-c levels. Meanwhile, a negative effect size indicates that decaffeinated coffee reduced the HDL-c levels. Horizontal lines represent the 95% CIs. Diamonds represent the pooled effect size from the random effect analysis. CGA: chlorogenic acid, CI: confidence interval, GCE: green coffee extract, HCCGA: high CGA content, HDL-c: high-density lipoprotein-cholesterol, MCCGA: medium CGA content. The values ± 0.2, ± 0.5 and ± 0.8, represent small, medium, and large effect sizes, respectively. * CGA dose not specified [21,22,26,29,30,31,32,33,34].

Four studies with six trials were included in the meta-analysis to investigate the effect of decaffeinated coffee on serum HDL-c levels. The effect size of mean increases on HDL-c levels (with small to large effect size; d = 0.06 and d = −0.80) is shown in Figure 5. Five out of six types of decaffeinated coffees increased the HDL-c levels (treatment group favoured). The greatest effect size was observed with decaffeinated coffee containing 510.6 mg of CGA (normocholesterolaemic participants), with d = 0.43 (95% CI: 0.18, 0.68), followed by that observed with decaffeinated coffee containing 428, 780 (high CGA content, HCCGA), 369, and 420 mg of CGA (medium CGA content, MCCGA), with d = 0.20 (95% CI: −0.04, 0.44), 0.13 (95% CI: 0.05, 0.21), 0.12 (95% CI: 0.06, 0.18), and 0.06 (95% CI: 0.00, 0.12), respectively. Meanwhile, one trial showed a null effect of decaffeinated coffee on HDL-c levels (favoured control group). Decaffeinated coffee containing 510.6 mg of CGA (hypercholesterolaemic participants) showed an effect size of d = −0.80 (95% CI: −1.13, −0.47). The data showed a high level of heterogeneity, with *I*^2^ = 86.8%, and, hence, were analysed using random effects analysis. The meta-analysed pooled effect size of decaffeinated coffee was d = 0.08 (95% CI: −0.05, 0.20) (Figure 5).

Five studies with six trials that investigated the effect of GCE on HDL-c levels were included in the meta-analysis (Figure 5). GCE showed a small-to-large effect size on mean HDL-c levels, with d = 0.19 and 1.40. All GCE interventions increased the serum HDL-c levels (treatment group favoured). GCE containing 180 mg of CGA showed the greatest effect size (d = 1.40, 95% CI: 1.11, 1.69), followed by that containing 376 mg of CGA, 1000 mg of CGE (CGA dose not specified), 250 mg of CGA (administered along with ERBT), 250 mg of CGA, and 372 mg of CGA (with d = 0.66, 95% CI: 0.42, 0.90; d = 0.58, 95% CI: 0.33, 0.83; d = 0.50, 95% CI: 0.32, 0.68; d = 0.49, 95% CI: 0.31, 0.37; and d = 0.19, 95% CI: 0.05, 0.33, respectively). A high level of heterogeneity (*I*^2^ = 91.3%) was observed in these data; hence, they were analysed using random effects analysis. GCE showed a moderate pooled effect size with d = 0.62 (95% CI: 0.34, 0.90) (Figure 5).

#### 3.4.5. Effect of Coffee on SBP

The effect of coffee consumption on SBP was evaluated on a short-term (60–120 min) and long-term (8–24 weeks) basis. Three short-term intervention studies investigated the effect of coffee (caffeinated coffee (*n* = 1) and decaffeinated coffee (*n* = 2)) on SBP. Decaffeinated coffee was more effective than caffeinated coffee in reducing SBP, with a mean percentage reduction of 1.5% and 0.6%, respectively. Nine long-term trials investigated the effect of coffee on SBP (caffeinated coffee (*n* = 1), decaffeinated coffee (*n* = 5), and GCE (*n* = 3)). Among the different types of coffee, GCE was the most effective in reducing SBP, with a percentage reduction ranging from 2.1% to 9.8% (Table 1).

**Figure 6:**
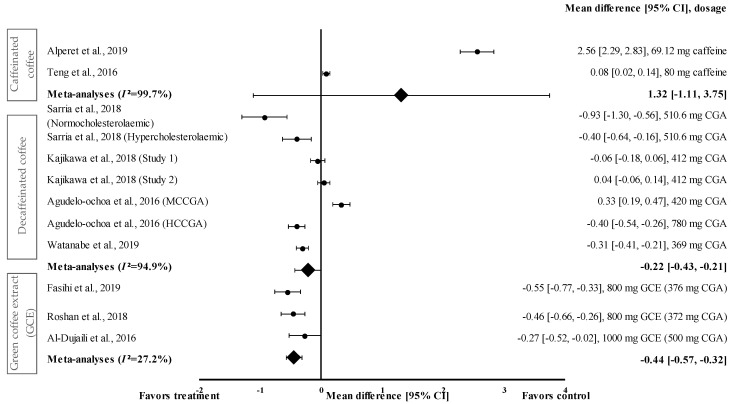
Forest plot showing the effect of caffeinated coffee, decaffeinated coffee, and GCE supplements on SBP, expressed as mean differences between the values obtained from the intervention and control groups. A negative effect size indicates that decaffeinated coffee and GCE supplements reduce SBP. Meanwhile, a positive effect size indicates that caffeinated and decaffeinated coffee increase SBP. Horizontal lines represent 95% CIs. Diamonds (*I*^2^ < 50%) represent the pooled effect size from fixed effects analysis. *I*^2^ >50% represents the pooled effect size from random effects analysis. CGA: chlorogenic acid, CI: confidence interval, GCE: green coffee extract, HCCGA: high CGA content, MCCGA: medium CGA content, SBP: systolic blood pressure. The values ± 0.2, ± 0.5 and ± 0.8 represent small, medium, and large effect sizes [22,23,24,25,26,27,30,31,33].

Figure 6 shows the meta-analysis of the effects of caffeinated coffee on SBP. Caffeinated coffee showed a small to very large effect size on mean SBP, with d ranging from 0.08 to 2.56. Caffeinated coffee increased SBP (favoured control group) compared to that in the control group. Caffeinated coffee containing 69.12 mg of caffeine showed the highest effect size (d = 2.56, 95% CI: 2.29, 2.83), followed by that containing 80 mg of caffeine (d = 0.08, 95% CI: 0.02, 0.14). A high level of heterogeneity (*I*^2^ = 99.7%) was observed in the data; hence, the data were analysed using random effects analysis. A large pooled effect size was reported for this meta-analysis, with d = 1.32 (95% CI: −1.11 to 3.75) (Figure 6).

Four studies with seven trials were included in the meta-analysis for investigating the effect of decaffeinated coffee on SBP. Decaffeinated coffee showed a small to large effect size on SBP reduction, with d values of 0.04 and -0.93 (Figure 6). In five out of seven interventions with decaffeinated coffee, SBP was reduced (treatment group favoured). Decaffeinated coffee containing 510.6 mg of CGA showed the greatest effect size (in normocholesterolaemic participants) (d = −0.93, 95% CI: −1.30, −0.53), followed by that containing 780 mg of CGA (HCCGA), 510.6 mg of CGA (in hypercholesterolaemic participants), 369 mg of CGA, and 412 mg of CGA (d = −0.40, 95% CI: −0.54, −0.26; d = −0.40, 95% CI: −0.64, −0.16; d = −0.31, 95% CI: −0.41, −0.21; and d = −0.06, 95% CI: −0.18, 0.06; respectively). Two trials showed a null effect on SBP reduction (favoured control group). Decaffeinated coffee containing 412 mg of CGA and 420 mg of MCCGA showed effect sizes with d values of 0.04 (95% CI: −0.06, 0.14) and 0.33 (95% CI: 0.19, 0.47), respectively. A high heterogeneity level of *I*^2^ = 94.4% was observed in the data; hence, the data were analysed using random effects analysis. Decaffeinated coffee showed a small pooled effect size on SBP with d = −0.22 (95% CI: −0.43, −0.21) (Figure 6).

GCE supplementation showed a small to moderate effect size in mean SBP reduction with d ranging from −0.27 to −0.55 (Figure 6). All GCE interventions reduced the SBP (treatment group favoured). GCE containing 376 mg of CGA showed the greatest effect size (d = −0.55, 95% CI: −0.77, −0.33), followed by that containing 372 and 500 mg of CGA (d = −0.46, 95% CI: −0.66, −0.26 and d = −0.27, 95% CI: −0.52, −0.02, respectively). A low level of heterogeneity (*I*^2^ = 27.2%) was observed in this meta-analysis and, hence, data were analysed using fixed effects analysis. The meta-analysis of GCE supplementation data showed a small pooled effect size with d = −0.44 (95% CI: −0.57, −0.32) (Figure 6).

#### 3.4.6. Effect of Coffee on DBP

The effect of coffee intake on DBP was evaluated on short-term (60–120 min) and long-term (8–24 weeks) bases, with the effect of caffeinated coffee investigated only on a short-term basis. Caffeinated coffee increased the DBP by 1.1% compared to the baseline (Table 1). Eight studies investigated the long-term effect of the consumption of coffee (caffeinated coffee (*n* = 1), decaffeinated coffee (*n* = 4), and GCE (*n* = 3)) on DBP. Decaffeinated coffee reduced the DBP by 7.3% to 3.3% compared to the baseline; however, in some studies, decaffeinated coffee also increased the DBP by 1.3% to 1.4% compared to the baseline (Table 1).

**Figure 7:**
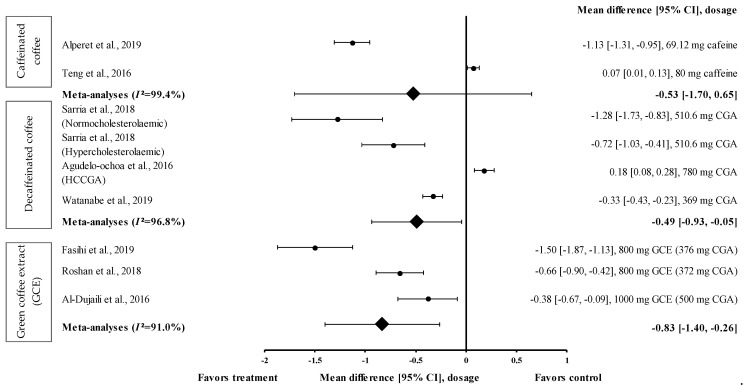
Forest plot showing the effect of caffeinated coffee, decaffeinated coffee, and GCE supplements on DBP, expressed as mean differences between the values obtained in the intervention and control groups. A negative effect size indicates that caffeinated coffee, decaffeinated coffee, and GCE supplements reduce DBP. Meanwhile, a positive effect size indicates that caffeinated and decaffeinated coffee increase DBP. Horizontal lines represent 95% CIs. Diamonds represent the pooled effect size from random effects analysis. CGA: chlorogenic acid, CI: confidence interval, GCE: green coffee extract, DBP: diastolic blood pressure, HCCGA: high CGA content, MCCGA: medium CGA content. The values ± 0.2, ± 0.5 and ± 0.8 represent small, medium, and large effect sizes [22,24,25,26,27,30,31,33].

Data from only two trials on caffeinated coffee were included in the meta-analysis (Figure 7). Caffeinated coffee containing 69.12 mg of caffeine reduced DBP (treatment group favoured) with an effect size of d = −1.13 (95% CI: −1.31, −0.95). Teng et al. showed that caffeinated coffee containing 80 mg of caffeine increased the DBP with an effect size of d = 0.07 (95% CI: 0.01, 0.13) [24]. A high level of heterogeneity (*I*^2^ = 99.4%) was observed in the data and, hence, the data were analysed using random effects analysis. The meta-analysis showed a moderate pooled effect size with d = −0.53 (95% CI: −1.70, 0.65) (Figure 7).

Three studies with four trials were included in the meta-analysis to investigate the effect of decaffeinated coffee on DBP. The effect size on mean DBP reduction ranged from small to very large, with d ranging from 0.18 to −1.28 (Figure 7). In three out of four trials on decaffeinated coffee, the mean DBP value was reduced (treatment group favoured). Decaffeinated coffee containing 510.6 mg of CGA (in normocholesterolaemic participants) showed the greatest effect size (d = −1.28, 95% CI: −1.73, −0.83), followed by that containing 510.6 mg of CGA (in hypercholesterolaemic participants) (d = −0.72, 95% CI: −1.03, −0.41) and 369 mg of CGA (d = −0.33, 95% CI: −0.43, −0.23). One trial showed a null effect on mean DBP reduction (favoured control group). Decaffeinated coffee containing 780 mg of CGA (HCCGA) showed an effect size of d = 0.18 (95% CI: 0.08, 0.28) [33]. The data from these studies showed a high level of heterogeneity (*I*^2^ = 96.8%) and, hence, were analysed using random effects analysis. Decaffeinated coffee showed a moderate pooled effect size on DBP (d = −0.49, 95% CI: −0.93, −0.05) (Figure 7).

Three intervention studies were included in the meta-analysis to investigate the effect of GCE supplementation on DBP. GCE showed a small to large effect size on DBP, with d = −0.38 and −1.50 (Figure 7). All interventions reduced DBP (treatment group favoured). GCE containing 376 mg of CGA showed the greatest effect size (d = −1.50, 95% CI: −1.87, −1.13), followed by that containing 372 and 500 mg of CGA (d = −0.66, 95% CI: −0.90, −0.42 and d = −0.38, 95% CI: −0.67, −0.09, respectively). The data showed a high level of heterogeneity (*I*^2^ = 91.0%); hence, they were analysed using random effects analysis. A large pooled effect size was observed with d = −0.83 (95% CI: −1.40, −0.26) (Figure 7).

## 4. Discussion

Caffeinated and decaffeinated coffees were the primary types of coffee used in the studies identified in this meta-analysis. Decaffeinated coffee contains 369–780 mg of CGA [22,23,30,31,32,33]. Only one study reported the CGA content of caffeinated coffee (45.4 mg) [28]. GCE is made from decaffeinated and unroasted coffee beans and, therefore, is classified as decaffeinated coffee [35]. GCE contains 180 to 500 mg CGA [20,21,26,27,28,29]. Fourteen studies showed an average risk of bias (score of 3 or above), whereas the remaining two studies showed a high risk of bias (score less than 3). GCE was administered to the participants as an extract. GCE supplementation effectively suppressed the MeTS parameters, namely waist circumference, TG and HDL-c levels, SBP, and DBP. Beverages containing decaffeinated coffee effectively reduced the FBG levels compared with the baseline. However, caffeinated coffee did not effectively improve the MeTS parameters, except for in terms of waist circumference, TG and HDL-c levels, and DBP.

This meta-analysis showed that GCE supplementation effectively improved anthropometric parameters, such as waist circumference. The pooled random effects analysis showed the small reducing effect on waist circumference (d = −0.39, 95% CI: −0.68, 0.10), albeit with a high level of heterogeneity (*I*^2^ = 93.0%) (Figure 2). Nevertheless, Fasihi et al. showed that supplementation with GCE containing 376 mg of CGA in capsule form for 8 weeks moderately reduced the waist circumference of participants, with d = −0.65 (95% CI: −0.89, −0.41) [30]. A recent review by Asbaghi et al. showed that, compared to the consumption of high-dose GCE for a short duration, the consumption of low-dose GCE (<400 mg of CGA/day) for 8 weeks effectively reduced body weight, waist circumference, and body mass index [36]. Green coffee beans are rich in CGAs such as 5-caffeoylquinic acid, one of the primary components of CGA that was shown to attenuate diet-induced obesity in mice [35]. The effect is modulated through the suppression of TG accumulation in the liver and the alteration of plasma adipokine levels, which subsequently downregulate adipogenesis-related genes and upregulate fatty acid oxidation-related genes [35,37].

A combination of resistance exercise and GCE supplementation was shown to considerably reduce the anthropometric parameters [38]. The findings of this meta-analysis showed that compared to only GCE supplementation (d = −0.07), the combination of supplementation with GCE (containing 250 mg of CGA) at a low dose and ERBT significantly reduced the waist circumference of participants (d = −0.45) [29]. Moghadam and Ganji showed that, compared to only GCE intake or concurrent training (CT), the ingestion of GCE (containing 420 mg of CGA) with CT (comprising of stretching and warm-up exercises, aerobic training, resistance training, and cool-down/running and stretching exercises) reduced the body weight and body mass index of women with obesity or women who are overweight [39].

Decaffeinated coffee intake reduced waist circumference, but less markedly than GCE supplementation. This could be attributed to the higher CGA content in GCE supplements than in decaffeinated coffee (liquid). The CGA content in coffee varies according to the food matrix; for instance, unroasted green coffee (capsule) has a higher CGA content than roasted coffee (10.2–21.1 g of CGA/100 g dry weight vs. 0.7–9.0 g of CGA/100 g dry weight, respectively) [40]. Reduced waist circumference was shown to be associated with improved glycaemic response [41]. A slight reduction in waist circumference (approximately 10% relative reduction) was associated with the reduction of FBG levels by 10 mg/dL. The findings of this review suggest that GCE and decaffeinated coffee help reduce waist circumference and may improve the glycaemic response.

Decaffeinated coffee containing 369 to 510.6 mg of CGA reduced the FBG levels to a greater extent than caffeinated coffee and GCE. Decaffeinated coffee showed a greater pooled random effect on FBG levels, with d = −0.81 (*I*^2^ = 96.4%), than caffeinated coffee and GCE (d = 0.17 and −0.30, respectively) (Figure 3). Decaffeinated coffee (liquid) containing 510.6 mg of CGA led to a greater effect size (d = −1.83) than decaffeinated coffee containing 369 mg of CGA (d = −0.06) [22,31]. This effect might be attributed to the different quantities of coffee in the two studies. In the first study, the participants consumed only one cup of coffee (containing 369 mg of CGA) per day, whereas in the second study, the participants consumed three cups of decaffeinated coffee (170.2 mg of CGA per cup) (breakfast, mid-day, and post-lunch) [22,31]. A previous study showed that phenolic metabolites, such as hydroxycinnamic acid, derived from CGA, are present in the bloodstream at relatively high concentrations for a longer period of time than caffeine, methylxanthines, and methylurics [42,43]. The intestinal absorption rate for CGA (33%) was lower than that for caffeic acid (95%) [44]. However, the mechanisms underlying the observed effects remain unclear. Hence, an understanding of how these metabolites affect FBG levels at the cellular level is warranted.

GCE reduced the FBG levels with an effect size of −0.30 (95% CI: −0.62, 0.03). However, it was less effective than decaffeinated coffee (Figure 3). This might be attributed to the different GCE doses (180–554 mg of CGA) and supplementation duration. Caffeinated coffee containing 69.12 to 554 mg of caffeine increased the FBG levels with an effect size of 0.17 (95% CI: 0.09, 0.25). The increase in the FBG levels caused by caffeinated coffee might be attributed to the varying caffeine contents in coffee. The findings of the study suggested that the reduction of glucose tolerance may have occurred in response to the increase in epinephrine levels after caffeine consumption. Desensitisation to the effects of epinephrine (via the downregulation of β-adrenergic receptors or the absence of epinephrine expression) could weaken the mechanism by which caffeine reduces glucose disposal [15]. GCE supplementation effectively reduced the TG levels and increased the HDL-c levels. The pooled random effects analysis showed the small and moderate effect sizes of the interventions on TG and HDL-c levels (d = −0.27, 95% CI: −0.43, −0.10 and d = 0.62, 95% CI: 0.34, 0.90, respectively). However, in individual studies, supplementation with GCE containing 180 to 376 mg of CGA in the capsule considerably reduced the TG level and considerably increased the HDL−c level, with d = −0.74 (95% CI: −0.99, −0.49) and d = 1.40 (95% CI: 1.11, 1.69), respectively. Mechanistically, this could be attributed to the stimulation of the hepatic peroxisomal proliferation-activated receptor-alpha (PPAR-α) by CGA present in the GCE. A previous study showed that activated PPAR-α plays a vital role in improving insulin sensitivity and inhibiting lipid synthesis in the liver [45]. Furthermore, CGA stimulates hepatic enzymes, such as fatty acid 3-hydroxy−3-methyl-glutaryl coenzyme A reductase, acyl-coenzyme A, and cholesterol acyltransferase, which subsequently increase the TG levels and promote cholesterol homeostasis [46].

This review also showed that GCE supplementation effectively reduced SBP and DBP. CGA was shown to reduce blood pressure and body weight by inhibiting 11β–hydroxysteroid dehydrogenase type 1 found in adipose tissues and the liver [47]. This enzyme is involved in the conversion of hormonally inactive cortisone into active cortisol, which reduces blood pressure and enhances weight loss.

GCE showed small and large pooled effect sizes on SBP and DBP, with d = −0.44 (95% CI: −0.57, −0.32; *I*^2^ = 27.2%) and d = −0.83 (95% CI: −1.40, −0.26; *I*^2^ = 91.0%), respectively (Figure 6 and Figure 7). However, in individual studies, supplementation with GCE containing 376 mg of CGA for 8 weeks exerted the strongest suppressive effect on both SBP and DBP, with d = −0.55 (95% CI: −0.77, −0.33) and d = −1.50 (95% CI: −1.87, −1.13), respectively. The effect was less pronounced after the short-term (2 weeks) consumption of GCE containing 500 mg of CGA. The short-term administration of GCE supplements, even at a high dose, may have been inadequate for reducing the SBP and DBP. 

A high level of heterogeneity was observed among the study data; hence, the results should be interpreted with caution. Additionally, subgroup and sensitivity analyses were not performed to identify the confounding factors that contributed to the MeTS outcomes.

## 5. Conclusions

Fourteen high-quality RCTs were included in this review, and the observation period in the studies ranged from 60 min to 24 weeks; the longer study periods were adequate for evaluating substantial changes in the MeTS parameters. The findings of this meta-analysis suggested that supplementation with GCE containing 180 to 376 mg of CGA for more than 4 weeks effectively reduced MeTS parameters, namely waist circumference (0.4% to 3.0%), FBG levels (0.8% to 14.8%), TG levels (2.2% to 11.3%), HDL-c levels (0.7% to 15.6%), SBP (2.1% to 9.8%), and DBP (4.7% to 6.7%). Supplementation with decaffeinated coffee containing 510.6 mg of CGA for more than 4 weeks effectively reduced the waist circumference (1.6%), FBG levels (4.1% to 4.9%), TG levels (1.2% to 4.6%), SBP (3.0% to 4.4%), and DBP (3.3% to 7.3%). GCE supplementation along with resistance exercise (i.e., ERBT) further enhanced the suppressive effect of GCE on MeTS parameters. However, the effects of GCE supplementation and decaffeinated coffee intake on MeTS outcomes varied depending on the dose administered and were independent of the intervention duration (60 min to 24 weeks). A more detailed intervention with a specific dose and a well-planned study design, with adjustment for dietary intake, physical activity, and other health outcomes, are needed to further confirm the outcomes reported in this review.

## Figures and Tables

**Table 1:** Summary of randomised controlled controlled trials (RCTs) included in systematic review (*n* = 16).

Author and Country	Study Overview	Change from Final vs. Baseline ^1^		Percent Reduction or Increment (%) $\left[\frac{Final-Baseline}{Baseline}\times 100\right]$		*p*-Value	Calculated Effect Size (ES)
		Intervention	Control	Intervention	Control		
Green coffee extract (GCE) (all in capsule form)							
Haidari et al., 2017 [20] Country: Iran	Subject: F: 64 (20–45 years), intervention: 30, control: 34 Study design: randomised, double-blinded, parallel Study duration: 8 weeks MeTS outcome: obesity (FMI: ≥8.7 kg/m^2^) Intervention: 400 mg/d GCE containing 180 mg CGA Control: 400 mg starch	FBG: −0.05 ± 0.08 mmol/L	FBG: −0.02 ± 0.07 mmol/L	FBG: −1.1	FBG: −0.4	FBG: 0.8 ^#^	FBG: −0.07
		TG: −0.04 ± 0.09 mmol/L HDL-c: 0.03 ± 0.01 mmol/l	TG: −0.06 ± 0.07 mmol/L HDL-c: −0.05 ± 0.004 mmol/L	TG: −2.2 HDL-c: 2.4	TG: −3.4 HDL-c: −3.9	TG: 0.07 ^#^ HDL-c: 0.15 ^#^	TG: −0.25 HDL-c: 1.40
Alhamhany et al., 2018 [21] Country: Iraq	Subject: M/F: 35 (20–55 years) Study design: randomised, crossover, single-arm Study duration: 6 weeks MeTS outcome: overweight/obesity (BMI: ≥25 kg/m^2^) Intervention: 1000 mg GCE Control: not available	FBG: −0.77 ± 0.27 mmol/L	N/A *	FBG: −14.8	N/A *	0.001 ^#^	−0.48
		TG: −0.19 ± 0.17 mmol/L HDL-c 0.14 ± 0.06 mmol/L	N/A *	TG: −10.7 HDL-c: 15.6	N/A *	TG: 0.061 ^#^ HDL-c: 0.03 ^#^	TG: −0.27 HDL-c: 0.58
Roshan et al., 2018 [26] Country: Iran	Subject: M/F: 43 (18–70 years), intervention: 21, control: 22 Study design: randomised, double-blinded, parallel Study duration: 8 weeks MeTS outcome: metabolic syndrome (according to IDF ^3^ guidelines) Intervention: 800 mg/d of GCE containing 372 mg CGA Control: 800 mg starch	WC: −2.40 ± 2.54 cm	WC: −0.66 ± 1.17 cm	WC: −2.3	WC: −0.6	WC: 0.009 *	WC: −0.42
		FBG: −0.28 ± 3.34 mmol/L	FBG: 1.63 ± 2.22 mmol/L	FBG: −3.3	FBG: 22.4	FBG: 0.036 *	FBG: −0.28
		TG: −0.07 ± 0.60 mmol/L HDL-c: 0.05 ± 0.22 mmol/L	TG: –0.25 ± 0.87 mmol/L HDL-c: 0.05 ± 0.09 mmol/L	TG: −10.6 HDL-c: 5.1	TG: −3.4 HDL-c: 5.3	TG: 0.439 * HDL-c: 0.923 *	TG: −0.18 HDL-c: 0.19
		SBP: −13.76 ± 8.48 mmHg DBP: −3.78 ± 7.30 mmHg	SBP: −6.56 ± 9.58 mmHg DBP: −6.13 ± 15.84 mmHg	SBP: −9.8 DBP: −4.7	SBP: −4.7 DBP: −6.9	SBP: 0.013 * DBP: 0.534 *	SBP: −0.46 DBP: −0.66
Beam et al., 2015 [28] Country: USA	Subject: M: 10 (19–34 years) Study design: randomised, double-blinded, crossover Study duration: 60–120 min MeTS outcome: healthy and overweight/class I obesity (BMI: 19.6–34.5 kg/m^2^) Intervention 1: 5 mg/kg BW of caffeine + 75 g dextrose Intervention 2: 10 mg/kg BW of GCE (5 mg/kg CGA) + 75 g dextrose Control: 5 mg/kg BW of dextrose + 75 g dextrose	*Intervention 1:* FBG: 0.60 ± 0.21 mmol/L *Intervention 2:* FBG: 0.40 ± 0.29 mmol/L	FBG: 0.90 ± 0.21 mmol/L	*Intervention 1:* FBG: 12.8, *Intervention 2:* FBG: 8.3	FBG: 19.1	N.S *	*Intervention 1:* FBG: 0.38 *Intervention 2:* FBG: 0.40
Al-Dujaili et al., 2016 [27] Country: Jordan	Subject: M/F: 16 (19–32 years) Study design: randomised, single-blinded, crossover Study duration: 2 weeks MeTS outcome: healthy and overweight/class I obesity (BMI: 18–35 kg/m^2^) Intervention: 1000 mg/d GCE containing 500 mg CGA and 25 mg caffeine Control: 25 mg tablet of caffeine	SBP: −4.60 ± 3.95 mmHg DBP: −4.30 ± 2.80 mmHg	SBP: −0.80 ± 4.09 mmHg DBP: −0.40 ± 3.20 mmHg	SBP: −3.9 DBP: −5.6	SBP: −0.7 DBP: −0.5	SBP: 0.001 * DBP: <0.001 *	SBP: −0.27 DBP: −0.38
Banitalebi et al., 2019 [29] Country: Iran	Subject: F: 60 (30–50 years), intervention 1: 15, intervention 2: 15, intervention 3: 15, control: 15 Study design: randomised, single-blinded, parallel Study duration: 8 weeks MeTS outcome: class I and II obesity (BMI: 30–40 kg/m^2^) *Intervention: 1* = Placebo (500 mg starch) + ERBT, *Intervention 2* = 500 mg GCE (~250 mg CGA) + ERBT, *Intervention 3* = 500 mg GCE (~250 mg CGA) Control: 500 mg starch	*Intervention 1:* WC: −2.57 ± 2.82 cm *Intervention 2:* WC: −2.54 ± 2.85 cm *Intervention 3:* WC: −3.10 ± 2.43 cm	WC: −1.00 ± 2.65 cm	*Intervention 1:* WC: −2.6 *Intervention 2:* WC: −2.5 *Intervention 3:* WC: −3.0	WC: −1.0	*Intervention 1:* WC: 0.001 ^#^ *Intervention 2:* WC: 0.001 ^#^ *Intervention 3:* WC: 0.001 ^#^	*Intervention 1:* WC: −0.60 *Intervention 2:* WC: −0.45 *Intervention 3:* WC: −0.07
Decaffeinated coffee							
		*Intervention 1:* FBG: −0.31 ± 0.18 mmol/L *Intervention 2:* FBG: −0.55 ± 0.23 mmol/L *Intervention 3:* FBG: −0.13 ± 0.17 mmol/L	FBG: 0.47 ± 0.34 mmol/L	*Intervention 1:* −5.9 *Intervention 2:* −10.5 *Intervention 3:* −2.5	9.3	*Intervention 1:* FBG: 0.001 ^#^ *Intervention 2:* FBG: 0.001 ^#^ *Intervention 3:* FBG: 0.071 ^#^	*Intervention 1:* FBG: −0.69 *Intervention 2:* FBG: −0.95 *Intervention 3:* FBG: −0.54
		*Intervention 1:* TG: −0.10 ± 0.18 mmol/L HDL-c: 0.07 ± 0.04 mmol/L *Intervention 2:* TG: −0.14 ± 0.15 mmol/L HDL-c: 0.08 ± 0.07 mmol/L *Intervention 3:* TG: −0.06 ± 0.16 mmol/L HDL-c: 0.03 ± 0.05 mmol/L	TG: −0.01 ± 0.17 mmol/L HDL-c: 0.01 ± 0.08 mmol/L	*Intervention 1*: TG: −5.6 HDL-c: 5.6 *Intervention 2:* TG: −7.7 HDL-c: 6.4 *Intervention 3:* TG: −3.6 HDL-c: 2.7	TG: −0.60 HDL-c: 0.80	*Intervention 1:* TG: 0.012 ^#^ HDL-c: 0.007 ^#^ *Intervention 2:* TG: 0.003 ^#^ HDL-c: 0.010 ^#^ *Intervention 3:* TG: 0.071 ^#^ HDL-c: 0.356 ^#^	*Intervention 1:* TG: −0.00 HDL-c: 0.55 *Intervention 2:* TG: −0.05 HDL-c: 0.50 *Intervention 3:* TG: −0.00 HDL-c: 0.49
Fasihi et al., 2019 [30] Country: Iran	Subject: M/F: 43 (25–50 years), intervention: 22, control: 21 Study design: randomised, double-blinded, parallel Study duration: 8 weeks MeTS outcome: metabolic syndrome (according to NCEP-ATP III ^2^ guidelines) Intervention: 800 mg/d GCE containing 376 mg CGA (capsule form) Control: 800 mg cellulose	WC: −1.40 ± 2.63 cm	WC: −0.60 ± 2.97 cm	WC: −1.3	WC: −0.5	WC: 0.14 *	WC: −0.65
		FBG: −0.73 ± 0.68 mmol/L	FBG: 0.20 ± 0.57 mmol/L	FBG: −8.4	FBG: 2.0	FBG: 0.25 *	FBG: −0.48
		TG: −0.27 ± 0.08 mmol/L HDL-c: 0.09 ± 0.06 mmol/L	TG: −0.12 ± 0.06 mmol/L HDL-c: −0.02 ± 0.06 mmol/L	TG: −11.3 HDL-c: 8.2	TG: −5.0 HDL-c: −1.9	TG: 0.09 * HDL-c: 0.02 *	TG: −0.74 HDL-c: 0.66
		SBP: −2.80 ± 2.02 mmHg DBP: −6.40 ± 1.96 mmHg	SBP: −1.20 ± 1.86 mmHg DBP: 2.00 ± 2.53 mmHg	SBP: −2.1 DBP: −6.7	SBP: −0.9 DBP: 2.1	SBP: 0.01 * DBP: 0 *	SBP: −0.55 DBP: −1.50
Watanabe et al., 2019 [31] Country: Japan	Subject: M/F: 142 (20–64 years), intervention: 72, control: 70 Study design: randomised, double-blinded, parallel Study duration: 12 weeks MeTS condition: overweight (BMI: 25–29 kg/m^2^) Intervention: instant regular coffee containing 369 mg CGA Control: instant regular coffee containing 35 mg CGA (liquid form) Volume: 180 mL	WC: −0.40 ± 0.85 cm	WC: −0.10 ±0.79 cm	WC: −0.4	WC: −0.1	WC: 0.012 *	WC: −0.08
		FBG: −0.04 ± 0.09 mmol/L	FBG: 0.09 ± 0.07 mmol/L	FBG: −0.8	FBG: 1.8	0.545*	−0.06
		TG: −0.06 ± 0.12 mmol/L HDL-c: 0.01 ± 0.06 mmol/L	TG: 0.19 ± 0.17 mmol/L HDL-c: 0.01 ± 0.06 mmol/L	TG: −4.3 HDL-c: 0.7	TG: −0.5 HDL-c: 0.7	TG: 0.965* HDL-c: 0.666 *	TG: −0.48 HDL-c: 0.12
		SBP: –6.7 ± 2.17 DBP: −5.2 ± 1.64	SBP: –3.9 ± 2.30 DBP: −3.8 ± 1.70	SBP: −5.1 DBP: −6.4	SBP: −2.9 DBP: −4.6	SBP: 0.812 * DBP: 0.395 *	SBP: −0.31 DBP: −0.33
Katada et al., 2018 [32] Country: Japan	Subject: M: 15 (20–60 years) Study design: randomised, double-blinded, crossover Study duration: 4 weeks MeTS outcome: healthy and overweight (BMI: 20.0–29.9 kg/m^2^) *Intervention 1:* CGA-enriched and HHQ-reduced coffee (CGA-HHQ (−): 428 mg CGA, 67 mg caffeine, 0.08 mg HHQ) (liquid form) *Intervention 2:* CGA-enriched and HHQ non-reduced coffee (CGA-HHQ (+): 382 mg CGA, 66 mg caffeine, 0.57 mg HHQ) (liquid form) Control: not available Volume: 185 mL	*Intervention 1:* TG: −0.03 ± 0.15 mmol/L HDL-c: 0.07 ± 0.13 mmol/L *Intervention 2:* TG: 0.08 ± 0.15 mmol/L HDL-c: −0.05 ± 0.12 mmol/L	N/A *	*Intervention 1:* TG: −2.8 HDL-c: 4.7 *Intervention 2:* TG: 7.5 HDL-c: −3.3	N/A *	N.S *	TG: −0.18 HDL-c: 0.20
Agudelo-ochoa et al., 2016 [33] Country: Colombia	Subject: M/F: 74 (20–60 years), intervention 1: 25, intervention 2: 24, control: 25 Study design: randomised, single-blinded, parallel Study duration: 8 weeks MeTS outcome: healthy and overweight (BMI: 18.5–29.9 kg/m^2^) *Intervention 1:* 420 mg CGA (MCCGA) (liquid form) *Intervention 2*: 780 mg CGA (HCCGA) (liquid form) Control: no coffee, no placebo Volume: 400 mL/d	*Intervention 1:* TG: 0.18 ± 0.20 mmol/L HDL-c: 0.01 ± 0.10 mmol/L *Intervention 2:* TG: 0.01 ± 0.17 mmol/L HDL-c: 0.01 ± 0.08 mmol/L	TG: 0.12 ± 0.26 mmol/L HDL-c: 0.05 ± 0.09 mmol/L	*Intervention 1:* TG: 13.5 HDL-c: 0.7 *Intervention 2:* TG: 0.7 HDL-c: 0.8	TG: 9.3 HDL-c: 3.8	TG: 0.09^#^ HDL-c: 0.16^#^	*Intervention 1:* TG: 0.10 HDL-c: 0.06 *Intervention 2:* TG: 0.03 HDL-c: 0.13
		*Intervention 1:* SBP: 1.00 ± 2.90 mmHg DBP: 1.00 ± 2.02 mmHg *Intervention 2:* SBP: −1.00 ± 2.67 mmHg DBP: 1.00 ± 2.09 mmHg	SBP: −2.00 ± 2.02 mmHg DBP: 0.0 ± 1.92 mmHg	*Intervention 1:* SBP: 0.9 DBP: 1.4 *Intervention 2:* SBP: −0.9 DBP: 1.3	SBP: −1.9 DBP: N.C	N.S ^#^	*Intervention 1:* SBP: 0.33 DBP: 0.00 *Intervention 2:* SBP: −0.40 DBP: 0.18
Sarria et al., 2018 [22] Country: Spain	Subject: M/F: 52 (18–45 years) Study design: randomised, single-blinded, crossover Study duration: 8 weeks MeTS outcome: normocholesterolemic (*n* = 25) (TC <200 mg/dL), hypercholesterolemia (*n* = 27) (TC > 200–240 mg/dL) Intervention: green/roasted coffee beverage containing 510.6 mg CGA/d (liquid form) Control: control drink (water/isotonic caffeine- and polyphenol-free drinks)	Normocholesterolemic: WC: 0.50 ± 0.40 cm Hypercholesterolemic: WC: −1.20 ± 0.69 cm	Normocholesterolemic: WC: 0.20 ± 0.40 cm Hypercholesterolemic: WC: −0.20 ± 0.69 cm	Normocholesterolemic: WC: 0.7 Hypercholesterolemic: WC: −1.6	Normocholesterolemic: WC: 0.3 Hypercholesterolemic: WC: −0.3	N.S *	Normocholesterolemic: 0.15 Hypercholesterolemic: −0.28
		Normocholesterolemic: FBG: −0.17 ± 0.03 mmol/L Hypercholesterolemic: FBG: −2.1 ± 0.03 mmol/L	Normocholesterolemic: FBG: 0.05 ± 0.03 mmol/L Hypercholesterolemic: FBG: −0.13 ± 0.03 mmol/L	Normocholesterolemic: FBG: −4.1 Hypercholesterolemic: FBG: −4.9	Normocholesterolemic: FBG: 1.2 Hypercholesterolemic: FBG: −3.0	FBG: 0.030 *	Normocholesterolemic: FBG: −1.83 Hypercholesterolemic: FBG: −0.66
		Normocholesterolemic: TG: −0.01 ± 0.02 mmol/L Hypercholesterolemic: TG: −0.04 ± 0.02 mmol/L	Normocholesterolemic: TG: −0.02 ± 0.02 mmol/L Hypercholesterolemic: TG: −0.03 ± 0.02 mmol/L	Normocholesterolemic: TG: −1.2 Hypercholesterolemic: TG: −4.6	Normocholesterolemic: TG: −2.5 Hypercholesterolemic: TG: −3.4	TG: 0.017 * *	Normocholesterolemic: TG: −0.12 Hypercholesterolemic: TG: −0.35
		Normocholesterolemic: SBP: −3.40 ± 0.61 mmHg DBP: −2.30 ± 0.34 mmHg Hypercholesterolemic: SBP: −5.20 ± 0.83 mmHg DBP: −5.60 ± 0.61 mmHg	Normocholesterolemic: SBP: −0.70 ± 0.59 mmHg DBP: −0.30 ± 0.31 mmHg Hypercholesterolemic: SBP: −3.60 ± 0.75 mmHg DBP: −3.50 ± 0.57 mmHg	Normocholesterolemic: SBP: −3.0, DBP: −3.3 Hypercholesterolemic: SBP: −4.4, DBP: −7.3	Normocholesterolemic: SBP: −0.6, DBP: −0.4 Hypercholesterolemic: SBP: −3.0, DBP: −4.6	SBP: 0.001 * DBP <0.001 *	Normocholesterolemic: SBP: −0.93, DBP: −1.28 Hypercholesterolemic: SBP: −0.40, DBP: −0.72
Kajikawa et al., 2018 [23] Country: Japan	Subject: M/F: 37 years Study 1: intervention 1: 10, intervention 2: 9 (53 ± 19 years) Study 2: intervention 1: 9, control: 9 (56 ± 15 years) Study design: randomised, single-blinded, crossover Study duration: 60–120 min MeTS outcome: borderline (SBP: 130–139 mmHg or DBP: 85–89 mmHg) or stage 1 hypertension (SBP: 140–159 mmHg or DBP: 90–99 mmHg) *Study 1: Intervention 1:* beverage A (CGA: 412 mg, HHQ: 0.11 mg, CAF: 69 mg), *Intervention 2:* beverage B (CGA: 373 mg, HHQ: 0.76 mg, CAF: 75 mg)*Study 2:* beverage AControl: beverage C (CGA: 0 mg, HHQ: 0.1 mg, CAF: 59 mg) (liquid form) Volume: 185 mL	*Study 1:* *Intervention 1:* TG: 60 min: 0.40 ± 0.26 mmol/L, 120 min: 0.65 ± 0.29 mmol/L *Intervention 2:* TG: 60 min: 0.30 ± 0.25 mmol/L, 120 min: 0.80 ± 0.33 mmol/L *Study 2:* *Intervention 1:* TG: 60 min: 0.21 ± 5.51 mmol/L, 120 min: 0.60 ± 5.53 mmol/L	TG: 60 min: 0.24 ± 1.01 mmol/L 120 min: 0.64 ± 1.19 mmol/L	*Study 1:* *Intervention 1:* TG: 60 min: 28.6, 120 min: 46.4 Intervention 2: TG: 60 min: 22.7, 120 min: 60.6 *Study 2:* *Intervention 1:* TG: 60 min: 15.3, 120 min: 43.8	TG: 60 min: 19.2 120 min: 51.2	*Study 1:* TG: N.S ^#^ *Study 2:* TG: N. S ^#^	*Study 1:* 60 min: 0.15 120 min: 0.05 *Study 2:* 60 min: 0.10 120 min: 0.07
		*Study 1:* *Intervention 1:* SBP: 60 min: 1.00 ± 4.02 mmHg, 120 min: 0.0 ± 3.84 *Intervention 2:* SBP: 60 min: −3.00 ± 3.66 mmHg, 120 min: −2.00 ± 4.02 mmHg *Study 2:* *Intervention 1:* SBP: 60 min: 1.00 ± 4.30 mmHg, 120 min: 0.0 ± 5.00 mmHg	SBP: 60 min: 2.00 ± 5.16 mmHg 120 min: −1.00 ± 5.16 mmHg	*Study 1:* *Intervention 1:* SBP: 60 min: 0.8, 120 min: N.C *Intervention 2:* SBP: 60 min: −2.3, 120 min: −1.5 *Study 2:* *Intervention 1:* SBP: 60 min: 0.8, 120 min: N.C	SBP: 60 min: 1.5 120 min: −0.8	*Study 1:* SBP: N.S ^#^ *Study 2:* SBP: N.S ^#^	*Study 1:* 60 min: 0.18 120 min: 0.06 *Study 2:* 60 min: 0.15 120 min: 0.04
Caffeinated coffee (all in liquid form)							
Teng et al., 2016 [24] Country: Malaysia	Subject: M/F: 104 (19–26 years), intervention: 53, control: 51 Study design: randomised, double-blinded, parallel Study duration: 60 min MeTS outcome: healthy and overweight/obesity (BMI ≥25 kg/m^2^) Intervention: instant coffee containing 82.2 mg caffeine Control: instant coffee containing undetectable decaffeinated coffee Volume: 250 mL	SBP: 0.65 ± 7.81 mmHg DBP: 0.62 ± 6.46 mmHg	SBP: −2.12 ± 6.28 mmHg DBP: −1.49 ± 4.91 mmHg	SBP: 0.6 DBP: 1.0	SBP: −1.8 DBP: −2.2	SBP: 0.05 * DBP: 0.64 *	SBP: 0.08 DBP: 0.07
Alperet et al., 2019 [25] Country: Switzerland	Subject: M/F: 126 (36–67 years), intervention: 62, control: 64 Study design: randomised, double-blinded, parallel Study duration: 24 weeks MeTS condition: overweight (BMI: 22.5–35.4 kg/m^2^) and non-insulin sensitive (HOMA-IR ≥ 1.30) Intervention: 100% instant Robusta coffee + 73.7% non-diary creamer (69.12 mg caffeine/d and 45.4 mg CGA/d) Control: 32.5% coloured non-dairy creamer + 67.5% non-dairy creamer (0 mg caffeine and CGA)	WC: −2.76 ± 0.14 cm	WC: 0.58 ± 0.13 cm	WC: −3.0	WC: 0.6	WC: 0.39 ^#^	WC: −0.70
		FBG: 0.30 ± 0.18 mmol/L	FBG: 0.11 ± 0.18 mmol/L	FBG: 6.3	FBG: 2.3	FBG: 0.09 ^#^	FBG: 0.16
		TG: −0.03 ± 0.19 mmol/L HDL-c: 0.04 ± 0.18 mmol/L	TG: 0.09 ± 0.18 mmol/L HDL-c: 0.00 ± 0.18 mmol/L	TG: −2.2 HDL-c: 3.4	TG: 7.5 HDL-c: N.C	TG: 0.69 ^#^ HDL-c: 0.18 ^#^	TG: −0.03 HDL-c: 0.01
		SBP: 1.36 ± 0.18 mmHg DBP: −0.01 ± 0.18 mmHg	SBP: −1.66 ± 0.18 mmHg DBP: −1.01 ± 0.18 mmHg	SBP: 1.1 DBP: −0.01	SBP: −1.3 DBP: −1.3	SBP: 0.33 ^#^ DBP: 0.16^#^	SBP: 2.56 DBP: −1.13

^1^ Values are mean + SD; N/A, not available; N.S, non-significance; N.C, no change; M, male; F, female; CAF, caffeine; DC, decaffeinated coffee; HHQ, hydroxyhydroquinone; CGA, chlorogenic acid; GCE, green coffee extract; MCCGA, medium chlorogenic acid content; HCCGA, high chlorogenic acid content; ERBT, elastic resistance band training; WC, waist circumference; FBS, fasting blood sugar; TG, triglyceride; HDL-c, high density lipoprotein cholesterol; SBP, systolic blood pressure; DBP, diastolic blood pressure. ^2^ NCEP-ATP III, National Cholesterol Education Program (Adult Treatment Panel III) guidelines (MeTS should have three of the following five features: waist circumference >102 cm for men and >88 cm for women, triglyceride >150 mg/dL, HDL-c <40 mg/dL for men or <50 mg/dL for women, blood pressure >130/85 mmHg or fasting blood glucose >100 mg/dL). ^3^ IDF, International Diabetes Federation guidelines (having central obesity (waist circumference >102 cm for men and >88 cm in women) with two of the following risk factors: triglyceride >150 mg/dL, HDL-c <40 mg/dL for men and <50 mg/dL for women, blood pressure >130/85 mmHg or fasting blood glucose >100 mg/dL). Effect size of ± 0.2, ± 0.5, and ± 0.8 represent small, medium, and large effect size, respectively. * *p*-value of treatment vs. control; ^#^ *p*-value of treatment vs. baseline.

**Table 2:** Summary findings on coffee types and doses used in RCTs.

Type of Coffee	No. of Study	Mode of Delivery	Dose
Caffeinated	*n* = 6	Powder	5 mg/kg BW–69.12 mg caffeine *
		Liquid	80 mg caffeine *, volume: 250 mL
Decaffeinated (including green coffee extract, GCE)	*n* = 11	Liquid	369–780 mg CGA *, volume: 180–400 mL
	*n* = 9	Capsule/tablet	10 mg/kg BW–1000 mg GC * containing 180–500 mg CGA

* dose used was based on per person/day.

**Table 3:** Jadad scores of RCTs (*n* = 14).

Studies	Score Descriptions					
	Randomisation (Yes/No)	Appropriateness of Randomisation (Detail)	Blinding (Yes/No) ^a^	Appropriateness of Blinding	An Account of All Participants or Description of Withdrawal or Dropouts	Total Score
Haidari et al., 2017 [20]	1	1	1	N/A	N/A	3.0
Alhamhany et al., 2018 [21]	1	N/A	N/A	N/A	N/A	1.0
Roshan et al., 2018 [26]	1	1	1	1	1	5.0
Al-Dujaili et al., 2016 [27]	1	1	0.5	N/A	1	3.5
Beam et al., 2015 [28]	1	N/A	1	N/A	1	3.0
Banitalebi et al., 2019 [29]	1	1	1	1	1	5.0
Fasihi et al., 2019 [30]	1	1	1	N/A	1	4.0
Watanabe et al., 2019 [31]	1	1	1	1	1	5.0
Katada et al., 2018 [32]	1	N/A	1	1	1	4.0
Agudelo-ochoa et al., 2016 [33]	1	N/A	0.5	N/A	1	2.5
Sarria et al., 2016 [22]	1	N/A	1	N/A	1	3.0
Kajikawa et al., 2018 [23]	1	N/A	0.5	1	1	3.5
Teng et al., 2016 [24]	1	1	1	1	1	5.0
Alperet et al., 2019 [25]	1	1	1	1	1	5.0

^a^ double blinded = 1 point; single blinded = 0.5 point; N/A: not available.

## Data Availability

Not applicable.
